# Risk factors of premature ejaculation and its influence on sexual function of spouse

**DOI:** 10.1186/s12610-020-00120-6

**Published:** 2021-02-18

**Authors:** Hu Li, Pan Gao, Jingjing Gao, Xu Wu, Guodong Liu, Yutian Dai, Hui Jiang, Xiansheng Zhang

**Affiliations:** 1 The First Affiliated Hospital of Anhui Medical University, Hefei, Anhui China; 2 Nanjing Drum Tower Hospital, Medical School of Nanjing University, Nanjing, Jiangsu China; 3 Department of Reproductive Medicine Center, Peking University Third Hospital, Beijing, 100191 China; 4 Department of Andrology, Peking University Third Hospital, Beijing, 100191 China

**Keywords:** Premature ejaculation, Female sexual dysfunction, Female sexual function index, Risk factors, Ejaculation précoce, Dysfonction sexuelle féminine, Index de la Fonction sexuelle féminine, Facteurs de Risque

## Abstract

**Background:**

Premature ejaculation (PE) is a multifactorial problem with a complicated aetiology that has detrimental effects on female partners’ sexual function. However, there is a lack of studies on the relationship between the factors related to PE and female sexual dysfunction (FSD) in China. We aimed to identify and explore the relationship between the factors associated with PE and FSD.

**Results:**

Ultimately, information was collected from 761 couples: 445 couples with PE complaints and 316 couples without PE complaints. The mean ages of the men with and without PE complaints were 36.29 ± 9.87 years and 31.48 ± 10.77 years, respectively. Female partners in the group with PE complaints reported lower total and subdomain female sexual function index (FSFI) scores, and approximately 65% of them were diagnosed with FSD (vs. control group: 31.96%). A PE duration of more than 14 months, a self-estimated intravaginal ejaculation latency time (self-estimated IELT) less than 2 min, a negative attitude towards PE problems, men’s introversion, and men’s depression were risk factors for FSD in the PE group.

**Conclusions:**

PE affects not only the patient himself but also the spouse. Comprehensive analysis reveals a clear relationship and interaction between female sexual function and PE. Moreover, in PE treatment, we should not ignore the occurrence of FSD and its impact and should emphasize the treatment of couples together.

## Résumé

**Contexte:**

L’éjaculation précoce (EP) est. un problème multifactoriel, avec une étiologie compliquée, qui a des effets néfastes sur la fonction sexuelle des partenaires féminines. Cependant, il existe un manque d’études en Chine portant sur la relation entre les facteurs liés à l’EP et la dysfonction sexuelle féminine (DSF). Nos objectifs étaient d’identifier et d’explorer la relation entre les facteurs associés à l’EP et la DSF.

**Résultats:**

Finalement, des informations ont été recueillies auprès de 761 couples: 445 couples avec plaintes d’EP et 316 couples sans plaintes d’EP. L’âge moyen des hommes avec et sans plaintes d’EP était respectivement de 36,29 ± 9,87 ans et 31,48 ± 10,77 ans. Les partenaires féminines du groupe avec plaintes d’EP ont obtenu des valeurs abaissées des scores, totaux et par sous-domaines, de l’indice de la fonction sexuelle féminine (IFSF), et environ 65% d’entre elles ont eu un diagnostic de DSF (pour 31,96% dans le groupe témoin). Une durée d’EP de plus de 14 mois, un temps intravaginal auto-estimé de latence d’éjaculation (IELT auto-estimé) de moins de 2 min, une attitude négative envers des problèmes d’EP, une introversion des hommes, et une dépression chez les hommes étaient des facteurs de risque de la DSF dans le groupe avec EP.

**Conclusions:**

L’EP affecte non seulement le patient lui-même, mais aussi la conjointe. L’analyse complète révèle une relation et une interaction claires entre la fonction sexuelle féminine et l’EP. En outre, dans le traitement de l’EP, nous ne devrions pas ignorer l’apparition de la DSF et son impact, et nous devrions mettre l’accent sur le traitement du couple.

## Background

Premature ejaculation (PE) is one of the most common sexual dysfunctions in men [1]. According to the International Society of Sexual Medicine’s (ISSM) definition of PE [2], PE involves three aspects: ejaculation that always or nearly always occurs prior to or within approximately 1 min of vaginal penetration from the first sexual experience (lifelong PE) or a clinically significant reduction in latency time, often to approximately 3 min or less (acquired PE); the inability to delay or control ejaculation; and negative personal consequences, such as distress, botheration, frustration, and/or the avoidance of sexual intimacy.

An increasing number of studies have suggested that PE is a multifactorial problem with a complex or undetermined aetiology [3, 4]. PE not only impacts the quality of life of male sufferers but also has detrimental effects on the mutual relationship and sexual satisfaction of spouses [5]. PE is closely related to female sexual dysfunction (FSD) and affects desire, arousal, lubrication, and orgasm [6, 7].

FSD can have a significant effect on women’s quality of life. Self-esteem, a sense of wholeness and relationships can be seriously and adversely affected, exacting a heavy emotional toll [8]. A previous study showed that the prevalence of adult female FSD in Beijing was 63.3%, higher than that in Europe (34.4%), the US (30%), and some Asian countries (43.8%) [9–11]. Although the incidence of FSD is high, little research has been done on the relationship between male sexual function and female sexual function. A community-based observational study found that female partners of men with PE complaints appeared to have greater sexual problems, including reduced satisfaction, increased distress and interpersonal difficulty, than partners of men without such complaints [12]. A large internet-based study also demonstrated that having a partner with PE complaints was a source of high levels of sexual distress [13]. With the development of the their problem, male patients might have a series of problems related to PE, and the responses of women also change gradually [14]. With the prolongation of the problem course, the patient’s condition deteriorates, and erectile dysfunction (ED) may occur and contribute to women’s complaints. Therefore, male sexual dysfunction may lead to the aggravation of FSD [15].

PE is closely related to female sexual function, and the conditions affect each other. At present, when encountering PE in the clinic, drug treatment is offered, but the role of female sexual function is neglected. This study mainly focuses on how male factors associated with PE affect female sexual function, which may reveal that FSD in patients with a partner with PE requires timely attention and emphasizes the treatment of couples together.

## Methods

### Subjects

From January 2018 to January 2019, an observational and cross-sectional field survey was conducted in the First Affiliated Hospital of Anhui Medical University in Anhui Province, China. Our research team established the database for this research gradually. All consecutive heterosexual couples who applied for sex therapy in the andrology outpatient clinic were invited to participate in the study. In addition, some couples were enrolled from our health examination centre. Subjects were divided into two groups, namely, a group with PE complaints and a group without PE complaints. PE complaints were evaluated by the Chinese version of the PE diagnostic tool (PEDT), as follows: total scores of 8 or less indicate no PE complaint; scores between 9 and 10 indicate an uncertain PE complaint; and scores of 11 or above indicate the presence of a PE complaint [16]. Therefore, couples with total scores of 8 or less were divided into a group with PE complaints; couples with scores of 11 or above were divided into a group without PE complaints. This study was reviewed and approved by the Anhui Medical University Research Subject Review Board.

Only subjects older than 18 years and in a stable heterosexual relationship of more than 6 months were eligible to participate in the study. A Chinese questionnaire [17, 18] and face-to-face guidance were used to collect the subjects’ information in this study. All men and their female partners had to comprehend and speak Chinese. The exclusion criteria were as follows: (a) patients who suffered from ED, as indicated by a score of < 22 on the Chinese version of the International Index of Erectile Function-5 (IIEF-5) [17]; (b) patients with oncological diagnoses, urological disorders, hypertension, diabetes mellitus and thyroid disorders; (c) patients taking other medications that could affect ejaculatory and/or psychological status, such as serotonin reuptake inhibitors or tricyclic antidepressants; (d) patients whose female partners had psychiatric, gynaecologic and systemic diseases that may affect sexual function or were taking antidepressants that affect sexual function; or (e) patients who visited the doctor without their female partner.

### Study design

Before study enrolment, all subjects were informed about the procedure of the survey. Those who participated in this study were asked to provide written consent. In addition, since several subjective and sensitive personal questions were included in the study, a pre-survey was administered to a small sample (*N* = 30) to modify the originally designed items to ensure that the questionnaire was comprehensive and easily understood.

The survey was conducted by professional andrology doctors, who provided face-to-face guidance. Before the study, the doctors were trained and had a good understanding of the process and purpose of this research. The questionnaires were completed by both men and women in the waiting room immediately following an initial interview. All subjects were required to complete a questionnaire that captured the following data: (i) demographic information (e.g., age, body mass index [BMI], education level, and employment status); (ii) duration of PE and medical and sexual histories (e.g., sexual desire, sexual frequency per month); (iii) self-estimated intravaginal ejaculation latency time (self-estimated IELT) (time from the start of vaginal insertion to the start of intravaginal ejaculation); (iv) incidence of men’s negative psychology burden according to the Zung self-rating anxiety-depression scales [19, 20]; and (v) men’s traits and attitudes towards PE problems.

In this study, men’s traits were evaluated by the Chinese version of the Eysenck Personality Questionnaire (EPQ), which consists of 88 items [21]. The EPQ is used to assess the personality of the respondent. It presents to the respondent yes/no questions for each item and includes 4 factors: extroversion/introversion (E), neuroticism (N), psychoticism (P) and lie (L). A high score on the E scale indicates extroversion, while a low score indicates introversion. In addition, this study assessed men’s attitude to PE problems by asking the following question: what is your attitude toward PE problems? According to some related elements of patients’ answer, such as proactive solution, optimality, courage to face, confidence, or little/no influence, etc., we commented that participants had a positive attitude towards PE problems. If the patient’s answer involved some elements, such as passive acceptance, depression, avoidance, or lack of confidence, etc., we commented that participants had a negative attitude towards PE problems.

The Zung Self-Rating Anxiety Scale (SAS) is a 20-item self-report assessment device. When answering each item, the person indicates the degree to which each statement applies to them. Each question is scored on a Likert-type scale of 1 to 4. The total score is obtained by summing the scores of the 20 items. A standard cut-off score of 50 is usually used to diagnose anxiety [19]. The Zung Self-Rating Depression Scale (SDS) contains 20 items, and its design is based on the diagnostic criteria for depression. Subjects rate each item with regard to how they have felt during the past several days using a 4-point Likert scale. The raw sum score of the SDS ranges from 20 to 80, but the results are usually presented as the SDS Index, which is obtained by converting the raw score to a 100-point scale [20]. The reliability of these instruments (the SAS and SDS) was assessed with Cronbach’s alpha coefficient, and the internal consistency values were 0.81 and 0.79, respectively.

The female partner of the patient was also evaluated with the Chinese version of the female sexual function index (FSFI) [16], which has six dimensions (sexual desire, arousal, lubrication, orgasm, satisfaction and pain). The reliability of the FSFI was assessed with Cronbach’s alpha coefficient. The internal consistency of the FSFI was 0.83, demonstrating acceptable internal reliability. Scores range from 2 to 36, and women with a total score less than 26 are considered to have FSD [22].

### Statistical analysis

SPSS version 13.0 software (SPSS Inc., Chicago, IL, USA) was used to analyse the data. Descriptive statistics were used to summarize the characteristics of the subjects. The normality check of continuous variables was performed with the Shapiro–Wilk test. Data are expressed as the mean ± standard deviation or number (percentage) when appropriate. The independent t-test and chi-square test were used for intergroup comparisons. Multiple logistic regression analysis was used to evaluate the correlation between factors of PE and FSD. For all tests, a *P* value less than 0.05 was considered statistically significant.

## Results

Of 1097 couples who were consecutively recruited, 140 couples were excluded with the corresponding exclusion criteria. Information was ultimately collected from 761 couples, including 445 couples with PE complaints and 316 couples without PE complaints. Subjects discontinued the study for the following reasons: “withdrawal of consent” (*N* = 96), “incomplete information” (*N* = 57) and “other reasons” (*N* = 43). In the PE complaint group, the mean age and BMI score of the men were 36.29 ± 9.87 years and 24.85 ± 3.39 kg/m2, respectively. Those of their female partners were 34.26 ± 8.83 years and 22.25 ± 5.06 kg/m2. The detailed demographic information for all subjects is shown in Table 1.

**Table 1:** Demographic characteristics of men and their female partners

Factors	With PE complaints				Without PE complaints				***P1*** value	***P***2 value
	(***n*** = 445)				(***n*** = 316)					
	Men		Female partners		Men		Female partners			
**Age (years)**	36.29 ± 9.87		34.26 ± 8.83		31.48 ± 10.77		29.15 ± 9.86		*< 0.001*	< 0.001
**BMI (kg/m**2)	24.85 ± 3.39		22.25 ± 5.06		22.28 ± 3.02		21.19 ± 4.23		*< 0.001*	0.271
**Education level (n%)**									0.357	*< 0.001*
High school or less	282	63.37%	353	79.33%	189	59.81%	204	64.56%		
University graduate	163	36.63%	92	20.67%	127	40.19%	112	35.44%		
**Employment status (n%)**									0.734	*< 0.001*
Student /Unemployed	168	37.75%	262	58.88%	124	39.24%	95	30.06%		
Employed	277	62.25%	183	41.12%	192	60.76%	221	69.94%		
**Resident (n%)**									0.236	0.063
Urban	159	35.73%	137	30.79%	99	31.33%	77	24.37%		
Rural	286	64.27%	308	69.21%	217	68.67%	239	75.63%		

**Data were expressed as mean ± standard deviation or number (percentage), when appropriate**

***PE*** Premature ejaculation

The difference between the men with PE complaint and no PE complaint were showed in P1 value

The difference between the female partners with PE complaint and no PE complaint were showed in P2 value

The significant differences between the group with PE complaints and the group without PE complaints with respect to the frequency of sexual intercourse, self-estimated IELTs, men’s traits, incidence of men’s negative psychological burden and attitude toward PE problems are listed in Table 2. We found that men with PE complaints reported a lower frequency of sexual intercourse (*p* < 0.001) and shorter self-estimated IELT (*p* < 0.001). There was also a higher incidence of introversion, negative psychological factors (e.g., anxiety, depression) and negative attitude towards PE complaints in the PE complaint group than in the control group (*p* < 0.001).

**Table 2:** Associated factors and Outcomes of FSFI scores in men with and without PE complaint group

Factors	With PE complaints		Without PE complaints		***P*** value
	(***n*** = 445)		(***n*** = 316)		
**Duration of relationships (years)**	8.75 ± 3.26		9.28 ± 4.03		0.625
**Duration of PE complaint (months)**	14.25 ± 7.66		–		–
**Frequency of sexual intercourse (times/month)**	4.27 ± 2.89		7.02 ± 3.03		< 0.001
**Self-estimated IELTs (minutes)**	2.75 ± 1.29		4.02 ± 2.01		< 0.001
**Men’s traits (n%)**					< 0.001
Introversion	277	62.25%	172	54.43%	
Extroversion	168	37.75%	144	45.57%	
**Psychology burden (n%)**					
Men’ s anxiety	90	20.22%	28	8.86%	< 0.001
Men’ s depression	38	8.54%	11	3.48%	< 0.001
Men’ s sexual desire disorder	90	20.22%	41	12.97%	< 0.001
**Attitude toward to PE complaint (n%)**					
Positive	188	42.25%	–		–
Negative	257	57.75%	–		–
**The incidence of FSD (n%)**	291	65.39%	101	31.96%	< 0.001
**FSFI scores**					
Total scores	22.39 ± 3.45		26.72 ± 3.26		< 0.001
Sub-domain scores					
Sexual desire	3.25 ± 1.12		4.45 ± 1.08		< 0.001
Arousal	3.72 ± 1.21		4.23 ± 1.26		< 0.001
Lubrication	4.22 ± 0.95		4.54 ± 0.89		< 0.001
Orgasm	3.67 ± 1.02		4.20 ± 1.12		< 0.001
Satisfaction	2.98 ± 0.96		3.78 ± 0.97		< 0.001
Pain during sexual intercourse	4.55 ± 1.29		5.52 ± 1.64		< 0.001

**Data were expressed as number (percentage) or mean standard deviation (SD)**

**Difference between with PE complaint group and without PE complaint group assessed by Chi-square test or t-test, as appropriate**

***FSFI*** Female sexual function index, ***PE*** Premature ejaculation, ***IELT*** Intravaginal ejaculation latency time, ***FSD*** Female sexual dysfunction

In addition to the above factors, the FSFI scores of all the female partners were calculated. Compared with those in the group without PE complaints, female partners in the group with PE complaints reported lower total and sub-domain FSFI scores. In addition, based on the FSD diagnosis standard, approximately 65% of female partners were diagnosed with FSD, which was higher than the rate in the control group (31.96%, *p* < 0.001).

Further analysis (see Table 3) shows that duration of PE, self-estimated IELT, men’s negative attitude towards PE problems, men’s traits and negative psychological burden were strongly correlated with FSD. All elements mentioned in this study, including PE duration of more than 14 months, self-estimated IELT less than 2 min, negative attitude towards PE problems, and men’s introversion and depression, were risk factors for FSD in the group with PE complaints.

**Table 3:** Factors associated with female sexual dysfunction in PE complaint group

	FSD		
	Odds ratio	95% Confidence Interval	***P*** value
**Duration of PE more than 14 months**	2.02	1.12–3.98	0.015
**Self-estimated IELTs less than 2 min**	2.54	2.03–5.17	< 0.001
**Men’ s negative attitude toward to PE problem**	3.47	2.26–7.79	< 0.001
**Men’ s introversion character**	2.89	2.09–6.45	< 0.001
**Men’ s depression**	1.92	1.23–3.74	0.021

**Multiple logistic regression analysis was used to evaluate the correlation between factors with PE and FSD**

***PE*** Premature ejaculation, ***FSD*** Female sexual dysfunction, ***IELT*** Intravaginal ejaculatory latency time

## Discussion

PE is a complex medical condition with many influencing factors, both physiological and psychological [23]. These factors can affect different aspects of female sexual function to varying degrees and even lead to sexual dysfunction [24]. However, when treating patients with PE, we often overlook the female partner’s own sexual function.

In our study, we found that female partners in the group with PE complaints reported lower total and sub-domain FSFI scores than those in the group without PE complaints. In addition, based on the FSD diagnosis standard, approximately 65% of female partners were diagnosed with FSD, which was higher than the rate in the control group (31.96%, *p* < 0.001). Most studies have reported significantly poor sexual functioning in the female partners of men who suffer from PE compared with that in the female partners of men without PE [25]. Burri and Spector reported that female partners in the PE group had a 7.12–9.83 higher possibility of having sexual distress than those in the non-PE group [6].

PE has negative effects on different aspects of women’s functioning. Bronner et al. studied the correlation between PE and female vaginal penetration difficulties (VPDs) and found that female partners of men with PE were found to experience significantly more VPDs, and the intensity of pain was higher in females whose male partners presented PE [26]. From the female partners’ perspective, female stress is closely related to the vaginal ejaculation latency time of PE patients. In addition, PE leads to the breakup of relationships and destroys the quality of couples’ relationships and sexual satisfaction [27]. Our findings reflected the close relationship among self-estimated IELT, duration of PE and female sexual function more directly.

In addition, psychological factors were more likely to be a bridge between males with PE complaints and their female partners with FSD. Rowland et al. found that men’s distress was associated with female orgasm disorders [28]. We found that negative psychological burden (anxiety, depression, and decreased sexual desire) was a risk factor for FSD by analysing male factors and FSD. We believe that some negative emotions caused by PE might cause couples to reduce communication and even create sexual disharmony.

As far as the emotional burden was concerned, patients often felt embarrassed and ashamed when they could not satisfy their partners. Lee et al., in analysing the exchange of problems between PE patients and their partners in the Asia-Pacific region, found that PE patients did not dare to face their own condition and could hurt the feelings of their partners by not engaging in adequate communication [29]. Patients tend to feel inferior, anxious, angry and disappointed and sometimes depressed. Kempeneers et al. found that men experienced more distress and dissatisfaction related to PE than did their partners [30, 31]. From our research, we found that patients with PE complaints were more introverted, which might explain why men responded as described above. In addition, we also found that PE patients showed evasive and negative attitudes towards their own problems. More interestingly, males’ traits and attitudes towards PE problems were risk factors for FSD. These factors may reduce couples’ satisfaction with their sexual life and further affect female sexual function. Of course, more research is needed in the future to confirm this relation.

The mechanism of the relation between PE and FSD is not well understood. Studies have shown that the incidence of PE might increase when women have sexual dysfunction [32]. Female partners of men with PE reported significantly greater sexual problems, with reduced satisfaction, increased distress and interpersonal difficulty, and more orgasmic problems than partners of non-PE men [33]. Miller and Byers found that men reported a significantly longer ideal duration of intercourse than did their partners [34]. With both men and women desiring intercourse to last approximately twice as long as the self-reported length, it is conceivable that this may lead to distress, displeasure and ultimately to the purchase of sex-enhancing medication, as observed currently among Ghanaians [35]. Discordance and misperceptions within heterosexual couples may affect sexual function. Other studies suggested that pain and lubrication associated with vaginal penetration might affect the ejaculation time of male partners [24]. A study conducted by Rajkumar et al. showed that anxiety was a risk factor for PE, which may be a plausible mechanism for explaining either the onset or persistence of PE based on the influence of autonomic dysfunction on premature ejaculation [36]. Combined with the former study, we found that PE and related influencing factors can directly affect female sexual function. Female sexual function disorder may in turn affect male ejaculation time [13], which may create a vicious circle. The interaction between PE and FSD will not only lead to sexual dysfunction in couples but could also cause the relationship to break up.

Based on the study’s results, we concluded that a PE duration of more than 14 months, self-estimated IELT less than 2 min, negative attitude towards PE problems, and men’s introversion and depression were likely to be risk factors for FSD in the PE group. This study implies that in FSD, the risk factors for PE and FSD need to be considered together. It is of great significance to improve the effectiveness of PE treatment and to coordinate the sexual relationship between husbands and wives [37].

Nevertheless, this study has several limitations. First, the surveys were conducted by a professional, which carries some risks, as this method of data collection is prone to response bias. Second, we have not explored the effects of FSD on male PE from a female perspective, which requires us to conduct more detailed research in the next step. Third, we collected information mainly in a face-to-face mode, and there will be deviations with regard to sensitive questions, so further investigation is needed. Finally, questions regarding personality traits and attitude towards PE were posed directly to the patient, and this information was not collected in another way, which may make our results too one-sided.

## Conclusion

PE not only affects the patient himself but also his spouse. Our study found that a PE duration of more than 14 months, a self-estimated IELT less than 2 min, a negative attitude towards PE problems, and men’s introversion and depression were risk factors for FSD. According to our comprehensive analysis, we cannot ignore the relationship and interaction between female sexual function and PE. Due to the small number of related studies, further investigation is needed.

## Data Availability

Datasets supporting the conclusions of this article are available and can be requested from the corresponding author.

## Abbreviations

BMI: Body mass index
ED: Erectile dysfunction
EPQ: Eysenck personality questionnaire
FSD: Female sexual dysfunction
FSFI: Female sexual function index
IELT: Intravaginal ejaculation latency time
PE: Premature ejaculation
PEDT: Premature ejaculation diagnostic tool
SAS: The Zung Self-Rating Anxiety Scale
SDS: The Zung Self-Rating Depression Scale
VPD: Vaginal penetration difficulties
